# Adequacy of the Fogging Test in the Detection of Clinically Significant Hyperopia in School-Aged Children

**DOI:** 10.1155/2019/3267151

**Published:** 2019-08-05

**Authors:** João Esteves Leandro, Jorge Meira, Carla Sofia Ferreira, Renato Santos-Silva, Paulo Freitas-Costa, Augusto Magalhães, Jorge Breda, Fernando Falcão-Reis

**Affiliations:** ^1^Department of Ophthalmology, São João Hospital, Porto, Portugal; ^2^Department of Surgery and Physiology, Faculty of Medicine, University of Porto, Porto, Portugal; ^3^Department of Anatomy, Faculty of Medicine, University of Porto, Porto, Portugal

## Abstract

**Purpose:**

To evaluate the efficacy of the “fogging test,” performed with a +2 diopters (D) lens, in the exclusion of clinically significant hyperopia in school-aged children.

**Methods:**

We studied 54 children between 5 and 11 years of age, with 10/10 best-corrected bilateral visual acuity (VA) without significant degree of correction. VA was assessed in each eye with a “bilateral” +2 D sphere over-refraction followed by cycloplegic retinoscopy. The capacity of the test to detect hyperopia of ≥+2 D and ≥+1.5 D was evaluated by examining the respective receiver operating characteristic (ROC) curves and sensitivity and specificity values for different cutoff values of VA.

**Results:**

For the detection of hyperopia ≥+2 D, the area under the ROC curve (AUC) was 0.955 (*p* ≤ 0.001). The VA cutoff with best discriminative capacity was ≥5/10, with a sensitivity of 100%, specificity of 79%, positive predictive value (PPV) of 57%, and negative predictive value (NPV) of 100%. In respect of ≥+1.5 D hyperopia, the test capacity was lower (AUC = 0.832; *p* ≤ 0.001). The best VA cutoff was also of ≥5/10, with a PPV of 81% and a NPV of 85%.

**Conclusion:**

The accuracy of the test was high for the evaluation of ≥+2 D hyperopia but lower for ≥+1.5 D hyperopia. For the detection of ≥+2 D hyperopia, the VA cutoff of <5/10 may permit the exclusion of clinically significant hyperopia in selected children, without the need for cycloplegia. For the same cutoff, the PPV was low, meaning that in children with ≥5/10 VA cycloplegic refraction remains obligatory.

## 1. Introduction

Hyperopia of ≥ +2.00 D is very frequent in children between 5 and 15 years of age with a prevalence ranging from 2.1% to 19.3% in different studied populations. The prevalence decreases as age increases, meaning 5% at age 7, 2-3% between age 9 and 14, and around 1% at age 15, according to a meta-analysis [1].

Hyperopic children are often able to maintain good visual acuity due to their high accommodative capacity [2] This accommodation effort can overcome the refractive error to some degree but may cause symptoms, such as asthenopia, difficulty with focusing, and headaches [1] These children may also present abnormalities of associated visual functions such as accommodation, vergence, and stereopsis [3]. Narayanasamy et al suggest that certain levels of hyperopia can affect reading performance and general performance in school [4].

There is wide controversy among experts regarding the correction of low hyperopia in school-aged children. Robaei et al. [5] considered that hyperopia of less than 2 D does not need correction, describing prescriptions for refractive errors less than 2 D as “nonrefractive prescriptions.” While this criterion appears to be popular among pediatric ophthalmologists, the +1.5 D cutoff has also been used by a considerable number of experts for the correction of asymptomatic children [6]. In patients with associated symptoms such as asthenopia, some authors correct errors as low as 0.75 D [7].

The fogging technique with a +2.00 sphere is described in the Clinical Optics textbook of the American Academy of Ophthalmology [8] for the detection of overminused or underplussed corrections during subjective refraction. It consists of fogging the endpoint refraction in each eye with a +2.00 D sphere and, according to this source, if the patient sees better than the 20/200–20/100 Snellen lines, the refraction values should be reconsidered. Besides the fact that the cutoff of visual acuity is not precise, there is no mention of the suitability of this test in children [8].

While there are no recent data on the use of this fogging technique in the ophthalmology office, variations of the procedure have been used in the past as a screening tool for hyperopia in school-aged children [9, 10]. However, the fogging method used in these reports differ from that described above, and none of them gives a comprehensive description of sensitivity and specificity values for different thresholds of VA, consequently not effectively clarifying the utility of the test for the examination of children in a clinical setting [9, 10]. Less accessible methods like handheld refractometers have been studied more extensively in the past years [11]. However, cycloplegia is necessary to obtain accurate results in children, and sensitivity for the detection of significant hyperopia in noncycloplegic conditions is low [12].

In this study, we aimed to investigate the efficacy of the method to exclude and detect significant levels of hyperopia (≥2 D) and to preclude the requirement for cycloplegic refraction, in asymptomatic school-aged children, with bilateral decimal 10/10 visual acuity. We also investigated the best VA cutoffs to be used for this purpose.

## 2. Methods

A convenience sample of 54 children from 5 to 11 years of age were assessed between April and September 2017 and underwent a full ophthalmologic examination.

All children were sent for routine ophthalmologic examination by the family doctor. The study was conducted in a tertiary central hospital which receives children referenced by the family physician from several areas of the north of the country, which include city, suburban, and rural population. None had learning difficulties. The study protocol was carried out in accordance with the tenets of the Declaration of Helsinki and received local Institutional Review Board approval.

In all participants, VA was measured monocularly by the same pediatric ophthalmologist, with the use of a computerized Snellen chart (decimal notation), with 200 candela/m^2^ luminance, at a distance of 4 meters with dim room illumination. For illiterate participants, angular visual acuity was tested using a “tumbling-E chart,” with single letters. Children were included if there was no relevant ocular pathology, and the decimal VA was of 10/10 in each eye with or without correction (that was no greater than 0.75 D of spherical equivalent) and no significant anisometropia (greater than 1.00 D). The fogging test was then performed and consisted of VA measurement with the same chart and conditions, with both eyes open and with +2 D lenses fitted into the spectacle frames. The child was given a few minutes with the +2 D lenses in place before VA testing, thus permitting the adequate control of accommodation.

After these measurements, cycloplegic refraction with retinoscopy was performed in all children by an expert pediatric ophthalmologist. Cycloplegia was induced with 3 drops of cyclopentolate hydrochloride 1% in each eye at 10-minute intervals followed by measurement 45 minutes later. Quality of the cycloplegia achieved was verified through verification of pupil dilation equal to or greater than 6 mm.

Two thresholds for clinically significant hyperopia were established based on the unilateral highest positive spherical equivalent between eyes: ≥1.5 D and ≥2.0 D. If the fogging test was performed over a minor subjective correction, the spherical equivalent from this correction was subtracted from the cycloplegic result.

### 2.1. Statistical Analysis

Statistical evaluation was performed using the SPSS statistical software [13]. Quantitative variables are expressed as the median and interquartile range, and qualitative variables are provided with their frequency distributions. To select the statistical test for comparisons, the normality of the quantitative data was assessed using the Shapiro–Wilk test. Mann–Whitney *U* test was used for qualitative data due to nonnormal distribution of data. The chi-square test was performed for categorical variables comparison.

The validity of the fogging test to detect hyperopia of +1.5 D and +2 D as measured by cycloplegic refraction was estimated by receiver operating characteristic (ROC) curves, and the sensitivity and specificity values were evaluated for different thresholds of VA. The best cutoff points of visual acuity reflect the best discriminative ability of the test to distinguish between children with and without hyperopia of ≥1.5 D and ≥2.00 D, considering different tradeoffs between sensitivity and specificity. Correlation between hyperopia (spherical equivalent) and VA with *a* +2 D sphere was tested using a Pearson correlation coefficient.

Statistical significance was set at a *P* value less than 0.05.

## 3. Results

The median age of participants was 6 [6; 8], and there were 26 males (48%) and 28 females (52%). Thirty-two (59%) patients were 5 or 6 years old.

Forty-one patients (71%) attained 10/10 VA in both eyes with no correction, while thirteen (24%) needed minor correction in either eye, the median spherical equivalent being +0.25 D [−0.50; +0.25]. Twenty-nine patients (54%) were evaluated with an angular vision chart and twenty-five (46%) with a Snellen chart.

The median spherical equivalent of the most hyperopic eye, according to the cycloplegic retinoscopy, was +1.25 D [+0.75; +1.75 D] with a median residual astigmatic error of +0.00 D [0, +0.50 D]. The median difference in spherical equivalent between eyes was +0.25 D [0, +0.25 D]. Eleven participants (20%) had hyperopia of ≥ +2 D and 22 (41%) of ≥ +1.5 D. Of children aged 6 or less, 28% had ≥2.00 D hyperopia, compared to 14% of children older than 6 years; however, the difference was not statistically significant (*p*=0.320, chi-square).

### 3.1. Visual Acuity with the Fogging Test with a +2 D Sphere

The median VA of the total cohort with an over-refraction of +2 D was 4/10 [3/10, 6/10]. In patients with <1.5 D of hyperopia the median VA was 3/10 [2.5/10, 4/10], while in patients with +1.50 D to +1.75 D of hyperopia, it was 5/10 [2/10, 7/10] and 8/10 [6/10, 8/10] in patients with ≥2.00 D of hyperopia.

There was a strong relationship between increasing spherical equivalent hyperopia and increasing VA with a +2 D sphere (Pearson correlation 0.795, *p* < 0.001). The presence of uncorrected astigmatism, age, chart type, and correction did not substantially influence the median VA with the +2.00 D sphere for different groups of spherical equivalent hyperopia (Table 1).

### 3.2. ROC Curves for the Detection of ≥1.5 D and ≥2 D Hyperopia

The area under the ROC curve (AUC) for the detection of ≥+2 D hyperopia was 0.955 (*p* ≤ 0.001) and 0.832 (*p* ≤ 0.001) for ≥ +1.5 D hyperopia. The performance of the ROC curves in both hyperopia thresholds were not influenced by the presence of uncorrected astigmatism of ≥0.5 D, age, chart type, and use of correction (Table 2).

The VA cutoff with greatest discriminative ability for detecting ≥ +2 D was ≥5/10, with a sensitivity of 100%, specificity of 79%, PPV of 57%, and NPV of 100%. For hyperopia of ≥1.5 D, the best cutoff was also ≥5/10, presenting a sensitivity of 77%, specificity of 88%, PPV of 81%, and NPV of 85%. For ≥+2 D hyperopia, the ≥8/10 cutoff presented a satisfactory specificity (95%) and NPV (93%), at the cost of a lower sensitivity (75%) and PPV (82%). The sensitivity and specificity of different thresholds of VA with the fogging test that allow the disclosure of refractive errors under cycloplegia of ≥ +2 D and ≥ +1.5 D are shown in Table 3.

## 4. Discussion and Conclusions

In the present study, we have examined a group of school-aged children in order to test the capacity of the fogging test with a +2 D sphere to detect or exclude clinically significant hyperopia. Cycloplegic refraction is the gold standard for measuring refractive errors in children [14–16]; however, it takes time, has costs and may cause significant discomfort to the patient. Therefore, a method that could reliably abbreviate its use in a considerable proportion of school-aged patients would be of great value.

Our study suggests that the fogging test may be adequate to exclude ≥+2 D hyperopia for a VA threshold of <5/10; however, various practical considerations should be taken in count.

First, it must be confirmed that patients tested by this method have 10/10 VA in each eye without significant correction or anisometropia, thus ensuring that both eyes of all patients have the same baseline VA prior to testing. High astigmatism can affect VA for a certain refractive error, in particular for low spherical values [17]; however, as median residual astigmatism in our cohort may be considered negligible, it did not significantly affect VA for different hyperopia values (Table 1).

In addition, children must be provided sufficient time to perform the fogging test, as it has been suggested that up to 10 or 15 minutes may be necessary for optical fogging to efficiently control accommodation [18]. In the present study, we did not define a specific minimum duration for testing as periods of 15 minutes are difficult to be attained in a normal consultation and would reduce the advantages of this method when compared to cycloplegia. Even using 10 or 15 minutes to efficiently control accommodation, it is still a gain of time compared to the 40 minutes used in the cycloplegia. On the contrary, this time does not make it unfeasible to use this test as a form of screening outside the ophthalmology clinic, like in schools. We consider that reasonable accommodation control for VA measurements can be achieved in cooperative children by meticulous testing, beginning in the 1/10 line and gradually advancing to progressively smaller optotypes.

Latent error results in the underestimation of the hyperopic refractive state of a child by using noncycloplegic measurements [19] and can range from 0.1 to 2 D between studies [18, 20], with high latent errors being associated with higher levels of hyperopia [19]. A fogging lens of +2 D was demonstrated to provide a level of accommodation control comparable to cycloplegia in adults [21]; however, it was not shown to be as effective in a study with school-aged children [18]. Whether a fogging lens of higher positive power could reduce the latent error and consequently improve the accuracy of the test for the detection of hyperopia in school-aged children may be the subject of future studies.

In addition, latent error and the accommodative response itself were reported to be influenced by the type of target that is used [22]. Differences between the Snellen and angular vision charts could potentially elicit different accommodation effects in children when evaluated with a positive fogging lens. However there seems to be an appropriate interchangeability between the VA measurements with these two charts (Table 1).

Overall, a criterion of decimal VA of 5/10 or less provides excellent sensitivity for the exclusion of hyperopia of ≥ D which is the usual cutoff used by the authors in prescribing asymptomatic schoolchildren. The NPV is 100%. This means that if a child, who has a VA of 10/10, tests the fogging with a +2.00 D lens and present a visual acuity worse than 5/10, we can exclude clinically relevant hypermetropia. These results mean that we can confidently abbreviate cycloplegic refraction in these patients. In this manner, fogging with a +2.00 D lens can be a simple test to be used as a screening method in schools outside the ophthalmology clinic.

The performance of the test for ≥1.5 D hyperopia was not as accurate (Table 3); therefore, clinicians considering this threshold to be clinically significant should not rely on the fogging test with the +2 D sphere. However, it would be worthy to investigate the performance of the fogging test with different sphere powers for the detection of ≥1.5 D hyperopia.

We acknowledge some limitations of this study. The most relevant limitation was the fact that a convenience sample was used instead of a proper sample size calculation for sensitivity and specificity. In addition, the percentage of patients with hyperopia of ≥+2 D (20%) seems higher than what is commonly described in this age group [1]. This reflects a selection bias that is due to the exclusion of patients with significant ametropias. Moreover, we used a Snellen chart for the collection of data despite the fact that logMAR (log of the Minimum Angle of Resolution) charts are recognized to provide more reliable VA measurements [23]; however, the first still remains the most widely adopted chart in clinical practice.

Kholer and Stigmar [9], in a Swedish community-based screening of the early 1980s that was mainly performed by nurses, rejected the utility of the fogging test as a method for hyperopia screening in that context. However, substantial differences in methodologies were found with the present study. First, the authors did not report the vision acuity measurement conditions in the screening process that if not effectively controlled can markedly affect the VA results, as pointed above. Secondly, they included children with uncorrected visual acuities ranging from 7/10 to 10/10 and evaluated them with +1.5 D and +2 D spheres, preestablishing the 7/10 line for the detection of ≥2.5 D hyperopia, not testing other VA cutoffs or hyperopia degrees. For these thresholds, they reported a sensitivity of 87.5% and specificity of 91.8%, considering that the test was not suitable for hyperopia screening because of low PPV that would lead to an intolerably high overreferral rate. Williams et al. [10] also tested a similar method as a part of the vision screening program of 7-8 years in Rhondda Cynon Taff performed by school nurses. In their study, a +4 D sphere was used to test children with uncorrected 10/10 visual acuity; those able to see any of the optotypes of the Snellen chart were considered test failures and were referred to an orthoptist. Again, they preselected the cutoff of visual acuity for that sphere power and did not consider other alternatives. They reported a 36% false positive rate for the detection of hyperopia of ≥2 D; however, they did not investigate their false negative rate.

We considered that the present study adds valuable data to these reports performed in a community-based setting and designed for screening purposes.

In conclusion, the fogging test with a + 2.00 D seems to be a fast and reliable tool to exclude hyperopia of ≥+2 D when considering a decimal visual acuity of <5/10 and it may preclude the use of cycloplegic refraction in carefully selected and collaborative school-aged children. However, clinicians should keep in mind that the accuracy of the test is dependent on the VA measurement conditions and subject-related variables. Future studies with larger samples and using different variations of the method may further validate these results and refine the performance data of this simple and useful test.

## Figures and Tables

**Table 1:** Influence of different variables on median VA with the +2.00 D sphere for different groups of spherical equivalent hyperopia.

	Hyperopia <1.5 D			Hyperopia ≥1.5 D and <2.0 D			Hyperopia ≥2 D		
	%, *n* = 32	VA	*p* ^*∗∗*^	%, *n* = 11	VA	*p* ^*∗∗*^	% *n* = 11	VA	*p* ^*∗∗*^
*Astigmatism*									
<0.5 D	75	3/10	0.209	55	3.5/10	0.177	82	8/10	0.291
≥0.5 D	25	3.5/10		45	6/10		18	9/10	

*Age*									
≤6 years	53	3/10	0.607	55	4/10	0.070	82	8/10	0.618
>6 years	47	3/10		45	7/10		18	7/10	

*Chart*									
Angular	53	3/10	0.422	73	6/10	0.364	36	7/10	0.161
Snellen	47	3/10		17	4/10		64	8/10	

*Correction* ^*∗*^									
No	71	3/10	0.612	82	5/10	1.000	82	5/10	0.618
Yes	19	3/10		18	5/10		18	6/10	

Visual acuity (VA) is reported as median. ^*∗*^Optic correction (≤0.75 D). ^*∗∗*^Data were derived from the Mann–Whitney test. *P* < 0.05, statistical significance.

**Table 2:** Area under the ROC curve (AUC) for the hyperopia's thresholds of ≥+2 D and ≥+1.5 D for different groups according to the presence of uncorrected astigmatism, age, chart type, and use of correction.

	Astigmatism		Age		Chart		Correction^*∗*^	
	<0.5 D	≥0.5 D	≤6 years	>6 years	Angular	Snellen	No	Yes
*≥+2 D*								
AUC	0.967 *p* < 0.001	0.972 *p*=0.014	0.961 *p* < 0.001	0.947 *p*=0.015	0.972 *p* < 0.001	0.929 *p*=0.003	0.969 *p* < 0.001	0.886 *p*=0.093

*>+1.5 D*								
AUC	0.804 *p*=0.002	0.875 *p*=0.015	0.804 *p*=0.003	0.886 *p*=0.004	0.837 *p*=0.005	0.836 *p*=0.002	0.832 *p* < 0.001	0.833 *p*=0.064

^*∗*^Optic correction (≤0.75 D).

**Table 3:** Sensitivity, specificity, positive predictive value (PPV), and negative predictive value (NPV) expressed in %, for different thresholds of visual acuity with the fogging test for the detection of ≥ +2 D and ≥ +1.5 D spherical equivalent errors.

	2/10	3/10	4/10	5/10	6/10	7/10	8/10	9/10
*Hyperopia >2.00 D*								
Sensitivity	100	100	100	**100**	92	75	75	25
Specificity	7	29	60	**79**	86	93	95	100
PPV	24	29	41	**57**	65	75	82	100
NPV	100	100	100	**100**	97	93	93	82

*Hyperopia >1.50 D*								
Sensitivity	96	86	86	**77**	68	50	46	14
Specificity	6	28	69	**88**	94	97	97	100
PPV	41	45	66	**81**	88	92	91	100
NPV	67	75	88	**85**	81	74	72	63

## Data Availability

The data used to support the findings of this study are available from the corresponding author upon request.
